# Transcriptome profile of cup-shaped galls in *Litsea acuminata* leaves

**DOI:** 10.1371/journal.pone.0205265

**Published:** 2018-10-24

**Authors:** Tin-Han Shih, Szu-Hsien Lin, Meng-Yuan Huang, Chih-Wen Sun, Chi-Ming Yang

**Affiliations:** 1 Biodiversity Research Center, Academia Sinica, Nankang, Taipei, Taiwan; 2 Department of Horticulture and Biotechnology, Chinese Culture University, Shihlin, Taipei, Taiwan; 3 Department of Life Science, National Taiwan Normal University, Wenshan, Taipei, Taiwan; Fred Hutchinson Cancer Research Center, UNITED STATES

## Abstract

**Background:**

Insect galls are atypical plant tissues induced by the invasion of insects. Compared to the host leaf, gall tissues lose photosynthetic ability, but have higher soluble sugar content. Although the physiological and biochemical regulation of gall tissues have been demonstrated, the mechanism of genetic regulation has only been analyzed in few studies.

**Results:**

In this study, the transcriptome of cup-shaped galls and its host leaf were *de novo* assembled. Cellular functional enrichment and differentially expressed gene groups in the gall tissues were analyzed. The genes associated with primary metabolism, including photosynthesis, cell wall turnover, and sugar degradation, were expressed differently in galls and leaves. The examination of gene expression demonstrated that the genes involved in brassinosteroid synthesis and responses exhibited a remarkable modulation in cup-shaped galls, suggesting a potential role of steroid hormones in regulating gall development.

**Conclusions:**

This study revealed the genetic responses, including those involved in source-sink reallocation and phytohormone metabolism, of galls induced by a dipteran insect.

## Introduction

Plant and insect interactions may alter many physiological functions and metabolic activities such that plants can respond to the stresses induced by insects or their larvae. Insect galls are one of the adaptive strategies that plants use, wherein they keep insect larvae within a tumor-like tissue outgrowth, apart from normal plant leaves, stems, and other organs. Gall tissues can protect insect larvae from natural enemies and provide a suitable microenvironment [1]. The formation of galls could be induced by the differential distribution of phytohormones, especially the development-associated auxin and cytokinin, produced by either the plant itself or the invading insects or fungi [2, 3].

Not only a shelter, gall tissues are able to provide nutrients for the insect larvae. The access to a nutritious resource might be accomplished either by the insect itself or via the aid of biotrophic fungi, which physically opens a channel from the gall tissues to the vascular bundles [4]. Moreover, leaf gallers manipulate the source-sink dynamics of nutrients in gall tissues and induce sink characteristics, such as an increase in soluble sugar content and a reduction in gas exchange and photosynthetic abilities [5–7]. However, the detailed mechanism by which this source-sink reallocation is initiated is still unclear.

Recently, the alteration of cellular machinery in gall tissues has been studied via systematic biological approaches. Nabity *et al*. [8] first used a transcriptomic approach to demonstrate that the invasion of phylloxera in grape leaves induced the expression of genes related to cell wall synthesis and biotic defense signaling, whereas the expression of photosynthetic-associated genes was reduced. Examination of the transcriptome in flooded gum (*Eucalyptus grandis*) revealed changes in genotype and defense mechanisms that the tree developed to resist the oviposition of the gall wasp [9]. Comparison of transcriptomic and genotypic analysis between galled and ungalled Myrtaceae leaves revealed that the genes that respond to auxin were strongly expressed in galled leaves [10]. This study failed to detect responses of auxin synthesis-associated genes, which supported the hypothesis that exogenous phytohormones are introduced by the gallers. The introduction of another phytohormone, cytokinin, to modulate the transcriptional activities in leaves was also suggested in a transcriptomic study of the interaction between apple trees (*Malus domestica*) and a leaf-mining moth [11]. These studies revealed the genetic adaptive machinery of plant cells after gall induction, and also demonstrated the feasibility of studying gall formation via bioinformatics methods, especially via transcriptomic approaches.

The galls induced by the Cecidomyiidae (Order: Diptera) in *Litsea acuminata* leaves exhibit an interesting morphology that not only protrudes from the leaf surface, but also forms a cup-like shape [5]. The ultrastructure and physiological function of cup-shaped galls were characterized in our previous study [5]. In cup-shaped galls, the photosynthetic capacity and stomatal conductance were undetectable, although the chlorophyll fluorescence was only slightly lower than that of the host leaf. The source-sink reallocation and nutrient provisioning feature of cup-shaped galls were confirmed by their photosynthetic deficit and accumulation of soluble sugars and free amino acids. To investigate the underlying mechanism of cellular regulation, the transcriptome of cup-shaped galls was examined by next generation sequencing (NGS) and RNA-seq methods in the present study. The genes from the galls and host leaves of *L*. *acuminata* were *de novo* assembled and differential gene expression were analyzed by the reads per kilobase of exon model per million mapped reads (RPKM)-fold change acquired from RNA-seq results. The enriched and differentially expressed genes that participated in photosynthesis and sugar-related metabolism were analyzed, as well as the genes associated with phytohormones.

## Materials and methods

### Plant material

**Fig 1:**
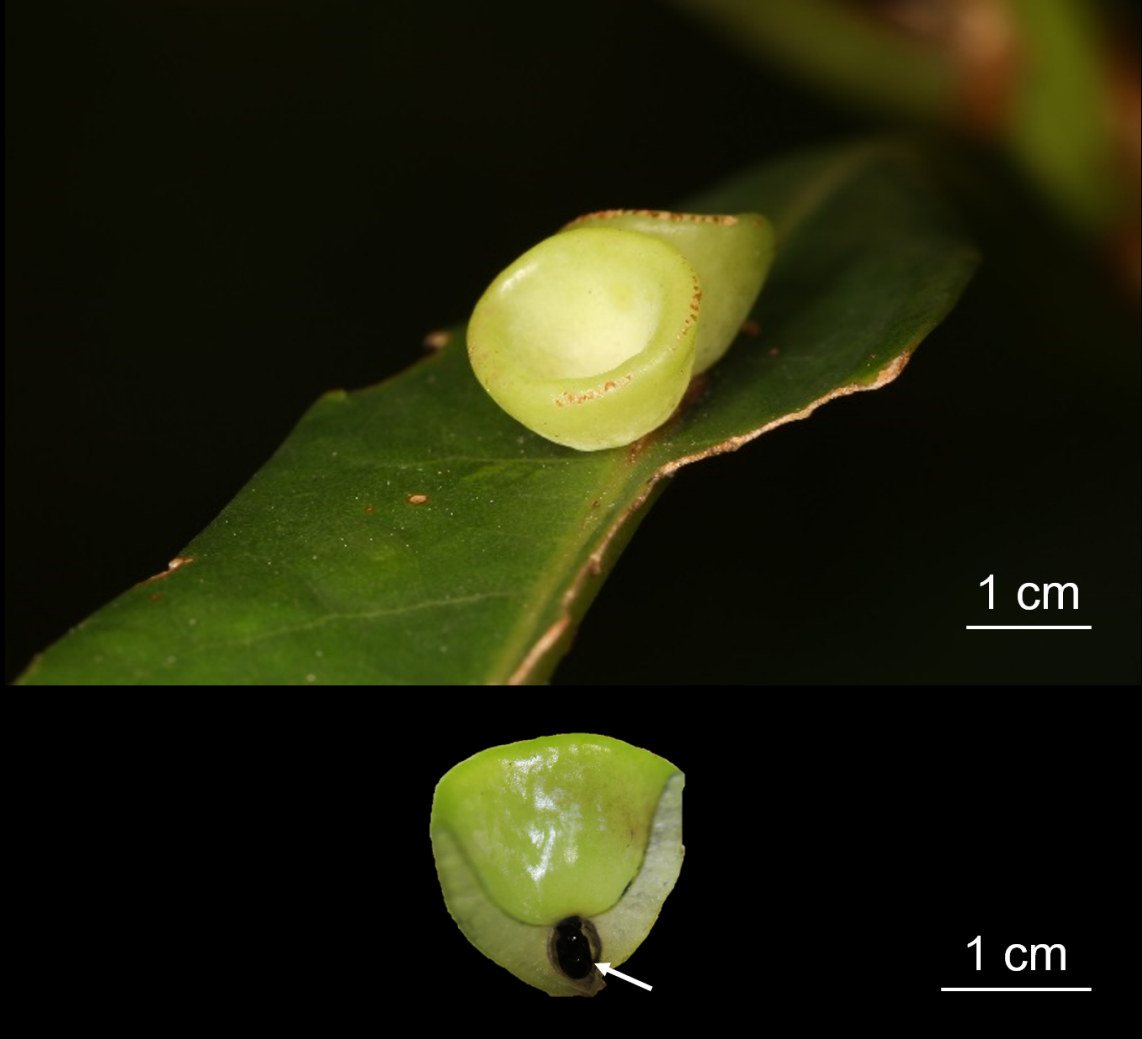
Image of cup-shaped gall in *Litsea acuminata*. A. Gall morphology B. Coronal anatomy of gall tissue.

The cup-shaped galls and their host leaves of *L*. *acuminata* (Fig 1) were collected from the Mt. Datun region, Yangmingshan National Park in Taipei, Taiwan (25.1861° N, 121.5252° E) with the permission of Yangmingshan National Park (Permission number 20180067). Detailed field growth and morphology of cup-shaped galls were described in our previous study [5]. The galls were sampled in late winter during maturation and immediately frozen using liquid nitrogen. The samples were stored at -80°C until use.

### RNA purification, cDNA library construction, and transcriptome sequencing

For transcriptome analysis and quantitative RT-PCR, total RNA of gall tissue and the host leaves (3 individual galls were pulled as one RNAseq sample as well as their host leaves) were prepared by the cetyltrimethylammonium bromide (CTAB)-based method [12]. For transcriptome sequencing, paired-end cDNA libraries for gall and host leaves were constructed. cDNA libraries were sequenced on an Illumina Hi-Seq platform (Illumina, San Diego, CA, USA). The ambiguous nucleotides, adapter sequences, and low-quality sequences were trimmed from the RAW reads. Sequencing and trim results were showed in Table 1. cDNA library construction and transcriptome sequencing were performed by a commercial service provider (Tri-I Biotech Inc., New Taipei City, Taiwan).

**Table 1:** Results of sequencing and trim.

Sample name	Number of reads	Avg. length	Raw data throughput (bp)	Number of reads after trim	Percentage trimmed
Host leaves	24,470,348	101	2,471,505,148	24,415,723	99.78%
Gall tissue	25,898,608	101	2,615,759,408	25,846,407	99.80%

### *De novo* assembly, BLAST, and RNA-seq

Paired-end reads were assembled using CLC bio’s *de novo* assembly algorithm (CLC Genomics Workbench v7.5, CLC bio, Denmark) to construct contiguous nucleotide sequences (contigs) with a minimum contig length of 200 (SRA accession ID: SRP132162). Non-coding RNA contigs were removed based on the BLAST of contigs against references (Rfam ver. 12.0). To obtain functional genes and remove redundant contigs, all contigs were BLAST analyzed against the proteins in the Plant Database (DB) with an identity setting of 0.5, according to the method described by Ono *et al*. [13]. The contigs with multiple hits were filtered and selected by the highest bitscore. All top hit contigs were considered functional genes in *L*. *acuminata*. The expression levels of genes were analyzed by the RPKM method via CLC Genomics Workbench v7.5. Genes with at least one RPKM value in one of two transcriptomes were defined as expressed, and only expressed genes were subjected to fold change analysis. Genes were aligned using CLC bio’s Align Contigs tool to find unique and shared genes between gall tissue and host leaves. The expressed genes were also searched in the *Arabidopsis* database (TAIR10) for specific annotation. The genes with the highest bitscore were kept when multiple *L*. *acuminata* genes were mapped onto duplicate *Arabidopsis* orthologs. Functional enrichment analysis was performed by MapMan (ver. 3.5.1, with PageMan integrated). Expressional differences were calculated by the fold change of RPKM. A two-fold RPKM value was used in MapMan visualization.

### Quantitative RT-PCR (qPCR)

One microgram of total RNA was extracted from gall tissue and host leaves for cDNA synthesis. cDNA synthesis was performed using a Transcriptor First Strand cDNA Synthesis Kit (Roche) and oligo(dT) as the primer. Primer sets for target and reference genes are listed in S5 Table. qPCR was performed using the StepOne Plus Real-Time PCR system (Thermo Fisher Scientific, Waltham, USA) with Roche FastStar Universal SYBR Green Master reagent (Roche). Relative gene expression values were presented as 2^-ΔCt^. ΔCt was calculated by subtracting the target gene Ct from reference gene Ct. Fold change of each gene in gall tissue was calculated by 2^-ΔCt^_gall_/2^-ΔCt^_leaf_. Fold change and statistical significance are shown in S4 Table. The relationship of fold changes obtained from qPCR and RNA-seq (normalized by RPKM) is plotted in S1 Fig.

### Statistical analysis

The significance of differences in relative gene expression (qPCR results) between gall tissues and host leaves was analyzed by Student’s *t*-test.

## Results

### *De novo* assembly, BLAST, functional annotation, and RNA-seq

**Fig 2:**
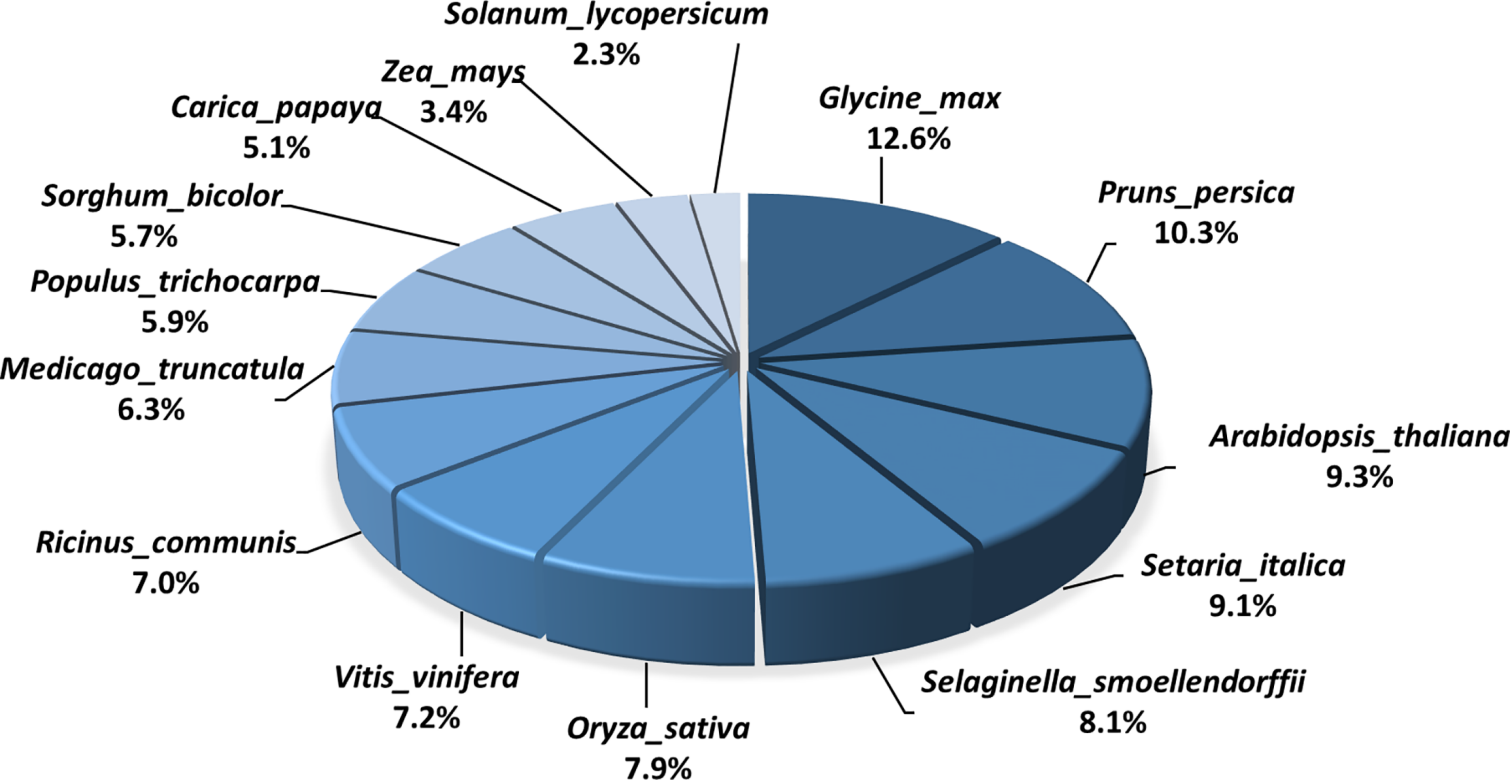
Species distribution of *Litsea acuminata* contigs BLAST searched against 14 plant species in the Plant Database.

**Fig 3:**
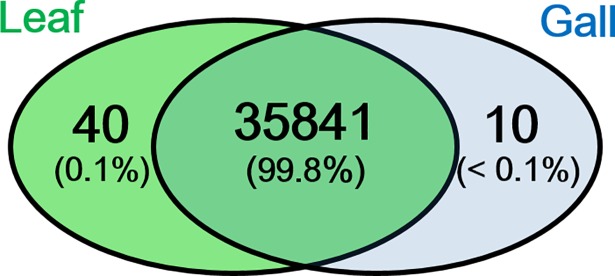
Venn plot displaying amount of shared and specific genes in gall tissue and the host leaf. Genes were the *de novo* assembled contigs after BLAST in Plant Database.

**Fig 4:**
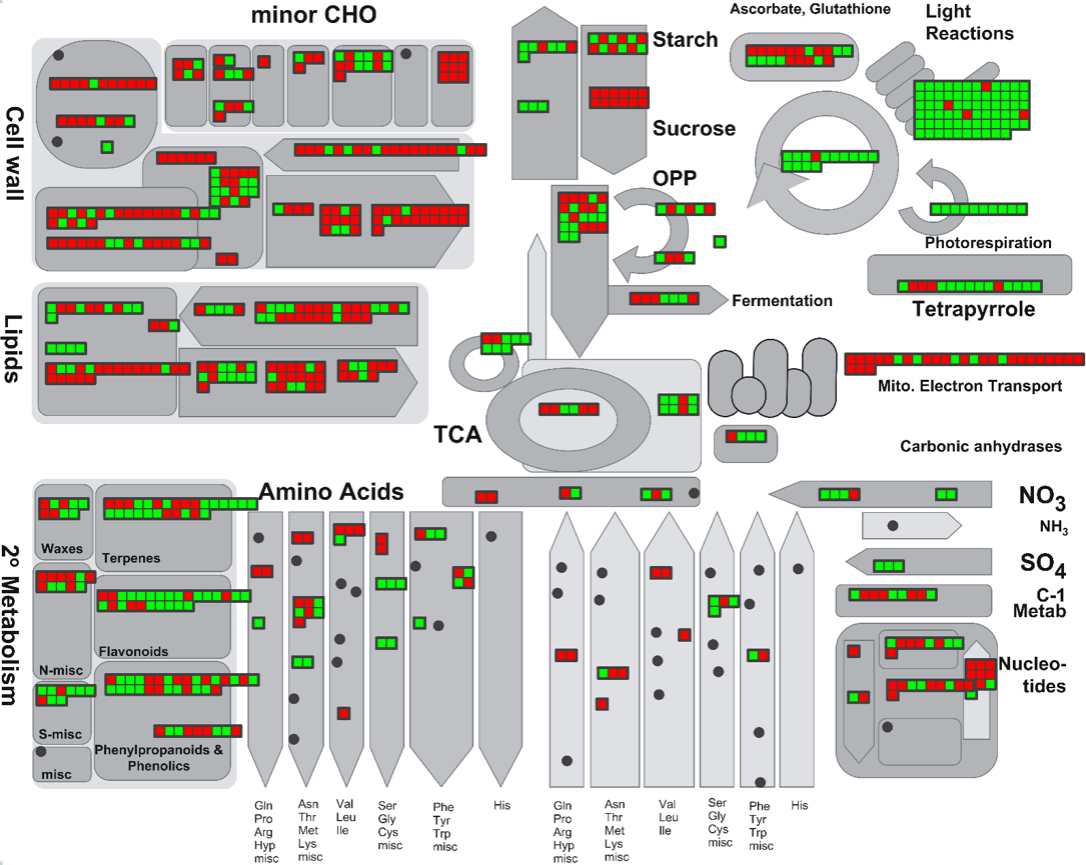
MapMan metabolic overview of genes with at least 2-fold expressional change in gall tissue. Red and green bins indicate up- and down-regulation, respectively.

*De novo* assembly of prepared gall tissues and host leaves produced 147,487 contigs when half of assembled contigs reached the length of 804 bps (N50 = 804) (Table 2). There were 106,155 contigs remaining after filtering by contig length ≥200 bp and read number ≥10. All contigs were subjected to homology searches to remove redundancies. Results showed that 35,892 contigs were obtained after BLAST search against the Plant DB (S1 Table). These contigs were regarded as the expressed genes of *L*. *acuminata* identified in the present study. Among 14 species BLAST searched, the genes of *L*. *acuminata* shared the highest identity (12.6%) with the genes from *Glycine max*, following by that of *Prunus persica* (10.3%) and *Arabidopsis* (9.3%) (Fig 2). In *L*. *acuminata*, over 99.8% of genes were shared between tissues of the galls and host leaves (Fig 3). To determine the genes that significantly contributed to cellular function, all genes were filtered by the minimum of RPKM = 1. The filtered genes were subsequently BLAST against the *Arabidopsis* database to obtain functional annotation. The results showed that 16,605 genes were annotated in the shared genes between galls and host leaves. In total, 5,909 annotated genes were had at least a 2-fold expressional change and were subsequently visualized by MapMan (Fig 4: Metabolic overview).

**Table 2:** *De novo* assembly and homological search in the Plant Database.

**De novo assembly**	
Number of de novo assembled contigs	147,487
Number of filtered contigs	106,155
N75 (bp)	536
N50 (bp)	804
N25 (bp)	1,721
**Plant DB cluster 50**	
Hit contigs	35,892
Unique hit contigs	19,036
Mutiple hit contigs	16,856
No hit	14,967

### Functional enrichment and differentially expressed genes in the cup-shaped galls

#### Photosynthesis and tetrapyrrole metabolism

**Fig 5:**
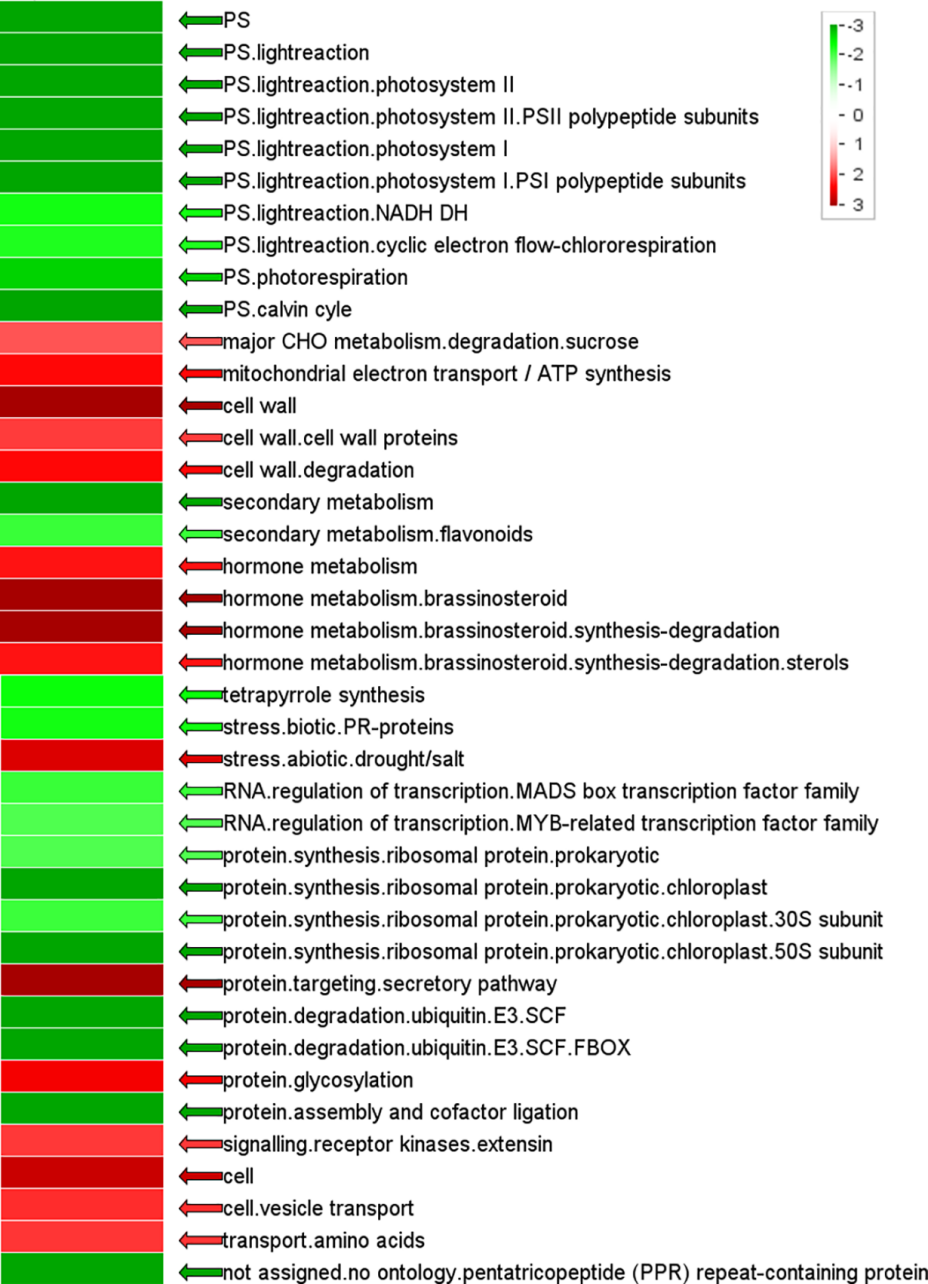
Enrichment of functional gene categories in gall tissue. Red and green indicate increased or decreased enrichment in gall tissue, respectively. Statistics were performed via the Wilcoxon test (with false discovery rate using the Benjamini-Hochberg procedure). Increased intensity of green and red represented increasing level of down- and up-regulation, respectively.

**Fig 6:**
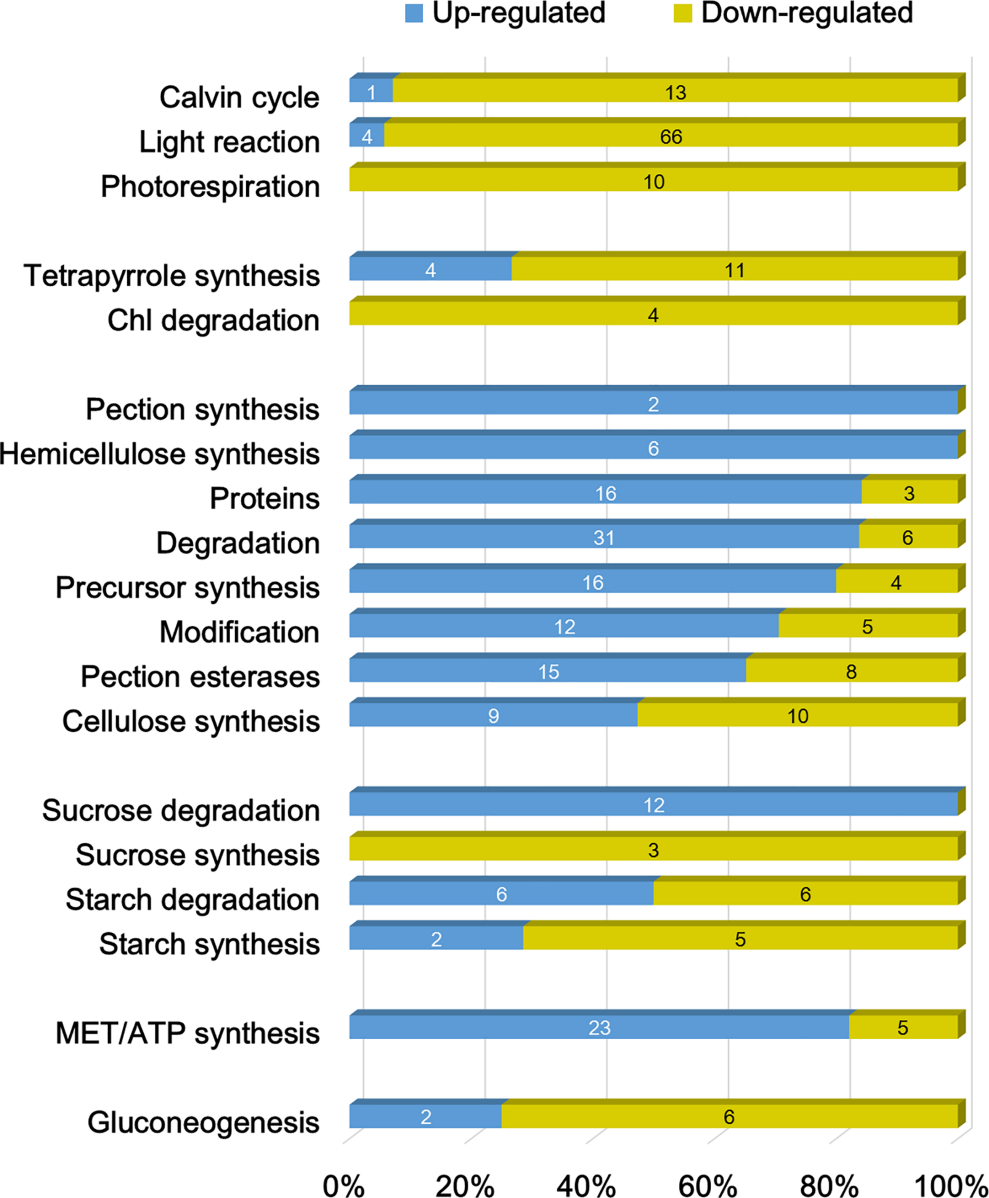
Ratio of up- and down-regulated genes among genes with at least 2-fold change in different functional groups. The groups associated with photosynthesis, tetrapyrrole, cell wall metabolism, major carbohydrate (CHO), mitochondria, and gluconeogenesis are demonstrated. Arabic number in each bar represents the gene number. Detailed descriptions of genes are listed in S2 Table.

The overview of the transcriptomic responses in gall tissues revealed that the function of the light reaction and Calvin cycle were significantly repressed (Figs 4 and 5), and approximately 95% of light reaction-associated genes that had at least a 2-fold expressional change exhibited a repressed pattern (Fig 6), including PSII core protein-coding genes *PSBA* (-5.2 fold) and *PSBC* (-2.6 fold) (S2 Table). Most of the genes related to ATP synthase (5/6) and cytochrome b6/f (6/6) were repressed in the galls. In the Calvin cycle, although most of the genes were down-regulated, the transcription of genes encoding the RuBisCo large or small subunit were unchanged or increased (*RBCS1A*: 12.8-fold), respectively.

In the cup-shaped galls, the function of tetrapyrrole synthesis and most genes involved in this pathway were also repressed (Figs 4 and 5). The genes encoding chlorophyllase I, chlorophyllase II, red chlorophyll catabolite reductase (RCCR), and pheophorbide a oxygenase (PAO), which are the key enzymes that participate in chlorophyll degradation, exhibited a >2 fold down-regulation (Fig 6, Chl degradation; S2 Table).

#### Mitochondria electron transport and gluconeogenesis

Results showed that the functions of mitochondrial electron transport and ATP synthesis might be enhanced in gall tissue; in particular, NADH dehydrogenase was enhanced. Most genes in complex I, III, and IV, as well as in F1-ATPase, exhibited greater transcription in galls than in host leaves. The gene encoding ATPase subunit 6 showed the highest up-regulation fold change (7.6-fold) in this functional category.

In the functional category of glyoxylate cycle (gluconeogenesis), 70% of annotated genes were expressed differentially between gall and host leaf tissues (Fig 6, Gluconeogenesis). The acetate-CoA ligase-coding gene was up-regulated 10-fold in gall tissue (S2 Table). However, the genes encoding enzymes that catalyze the later steps of the glyoxylate cycle were down-regulated, including isocitrate lyase (-13.5 fold), malate synthase (-9.3 fold), malate dehydrogenase 1 (-2.1 fold), and phosphoenolpyruvate (PEP) carboxykinase (-9.7 fold). Another gene coding for pyruvate, phosphate dikinase (PPDK), was also highly repressed in gall tissue (-15.3 fold).

#### Phytohormones and stress responses

**Fig 7:**
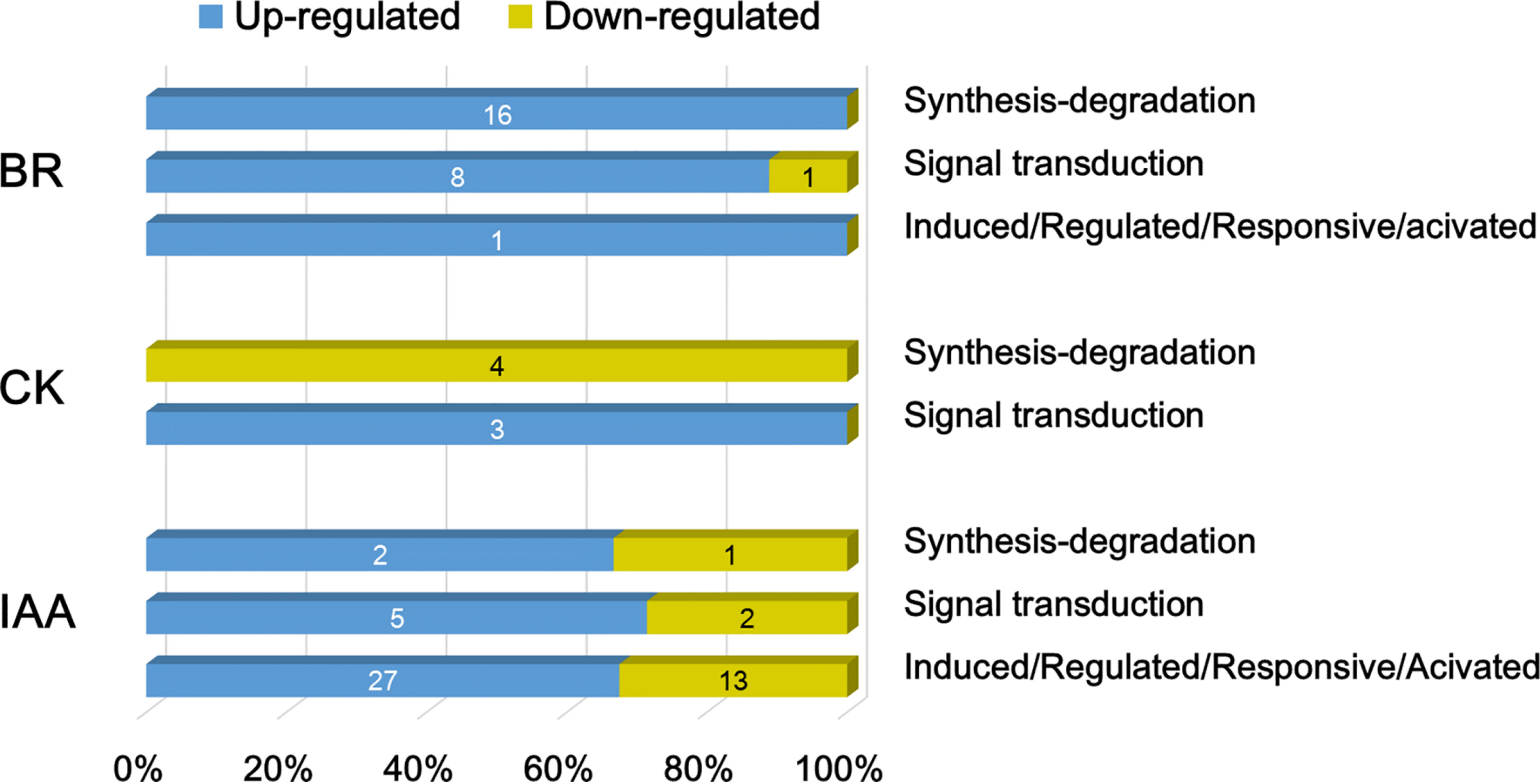
Ratio of up- and down-regulated genes among genes with at least 2-fold change in the categories and subcategories of brassinosteroid (BR), cytokinin (CK), and indole-3-acetic acid (IAA). Arabic number in each bar represents the gene number. Detailed descriptions of genes are listed in S3 Table.

Enrichment analysis of transcript amount showed that in gall tissue, the brassinosteroid-associated metabolism might be highly functional at the transcriptional level (Fig 5), exhibiting an up-regulation of the differentially expressed genes involved in brassinosteroid (BR) synthesis-degradation and the signaling pathway (Fig 7, S3 Table). The genes encoding squalene epoxidase (SQE3), steroid hydroxylase (BAS1), sterol methyltransferase (SMT3), and sterol delta7 reductase (DWF5) were expressed ≥4-fold in gall tissue. The function of two other important phytohormones, auxin (indole-3-acetic acid, IAA) and cytokinin (CK), were not enriched in the cup-shaped galls. However, it was shown that 50 genes participating in the biosynthesis and signal transduction of auxin were differentially expressed in gall tissue (Fig 7). The analysis of differentially expressed genes showed that the genes in CK synthesis/degradation were repressed in galls, whereas those related to the CK signaling pathway were up-regulated (Fig 7, S3 Table).

Gall tissue was enriched with genes responding to drought and salt-induced abiotic stress. Conversely, although the tissue was invaded by insects and fungi, genes responding to biotic stress were repressed in gall tissue, particularly the genes encoding pathogenesis-related (PR) proteins (Fig 5). The genes encoding respiratory burst oxidase protein (RBOH), which were demonstrated to mediate the infective responses though the production of reactive oxygen intermediates [14], exhibited highly increased expression (4 isoforms, 11.9–14 fold) in gall tissue (S2 Table).

#### Cell wall and carbohydrate (CHO) metabolism

Genes associated with cell wall metabolism were enhanced in gall tissue (Fig 5, S2 Table). Although the genes from the functional categories of cell wall synthesis (cellulose, hemicellulose, and pectin) were not statistically enriched in gall tissue, over 90% of these genes had unchanged or increased transcription levels. The genes involved in the function of cell wall degradation and modification, and pectin esterases also exhibited unchanged or increased ratios (over 88%). Notably, the expression of *PGIP1*, a gene induced by fungi that encodes a polygalacturonase that inhibits the enzymatic degradation of cell wall pectin [15], was up-regulated over 100-fold.

Among the genes involved in CHO metabolism, the genes encoding proteins related to sucrose degradation were highly expressed in gall tissues (Fig 5). All the differentially expressed sucrose synthesis-related genes were down-regulated, whereas those related to sucrose degradation were up-regulated.

An approximately 60-fold increased level of expression was observed for the invertase (β-fructosidase 3) coding gene (S2 Table). The gene encoding sucrose-phosphate synthase (a sucrose-degradation enzyme) was down-regulated ~28-fold. No gene related to sucrose synthesis within the CHO metabolism function was up-regulated. Furthermore, no gene related to sucrose degradation was down-regulated.

## Discussion

Although their morphology is distinct from that of leaves, gall tissue had a similar number of genes as did the host leaves, having 99.8% of expressed gene being identified as those found in leaves (Fig 3). This indicated that the invasion of insects only altered the regulatory responses of gene expression, and did not change the cellular processes related to organ proliferation. Current reliable methods were applied to reduce redundancy, such as searching the homology of reference genomes from related species, while performing the *de novo* assembly [16]. To date, however, *L*. *acuminata* have no related species that are phylogenetically close with a sequenced genome. The reprogrammed transcriptome profile of gall tissues in present study showed that physiological and biochemical changes were corresponded to those in previous gall studies, and the redundant gene-removing methodology (BLAST against Plant DB) executed in this study was feasible and reliable for uncovering the functional gene groups.

### Source-sink reallocation

In our previous research, the gall tissues in *L*. *acuminata* leaves were found to lose photosynthetic ability, as well as experience a reduction in chlorophyll and other photopigments [5]. Thus, it was suggested that the loss was an autotrophic characteristic. In the present study, we used the transcriptomic approach to reveal gene expression patterns of gall tissues and indicated that the loss of photosynthetic function was caused by the repressed expression of photosynthesis-associated (light and dark reaction) genes. Because the deficiency of photosynthesis and the photosystem occurred, it was reasonable to observe a decreased synthesis of tetrapyrroles, and the accompanied down-regulation of the chlorophyll-degradation function because the substrate (chlorophyll) was not synthesized in the gall. Nabity et al. [8] showed that the expression of photosynthesis-related genes was reduced in galls compared to undamaged leaves. The present study showed similar results in the comparison of gall tissue and the host leaves, suggesting that the host leaves remained as functional as undamaged leaves.

The reprogrammed expression of energy- or nutrient-associated genes in cup-shaped galls also indicated a characteristic shift from autotrophic to heterotrophic. The enrichment of the mitochondrial electron transport chain and the higher transcription of sucrose degradation-related genes in gall tissue suggested a requirement for energy (ATP) to maintain primary metabolism. The increased expression of sucrose degradation-associated genes, including invertase and hexokinase, might contribute to the elevated soluble sugar content, which was demonstrated in our previous study [5]. In the galls of grape leaves, it was found that the genes related to sucrose transport were increased and the carbon was derived from the leaf [8]. These results supported the idea that gall tissue might provide nutrients for insect larvae [5, 9].

The increased soluble sugar content in gall tissue might contribute to the structural formation. High content of glucose might increase the production of cellulose, thereby enhancing the biosynthesis of polysaccharides used in cell wall metabolism, a function that was enriched in gall tissue (Fig 5). Moreover, sugar could act as a signal for developmental and physiological responses to abiotic and biotic stresses [17]. The elevated sugar content in tissues was shown to repress photosynthetic activities and inhibit the expression of photosystem genes [18–20], which subsequently reprogrammed the source-sink partitioning in the leaf [17].

Although previous studies showed a down-regulation of tricarboxylic acid (TCA) cycle-related genes in gall tissues [8], the present study failed to detect a significant change in TCA function. Instead, the expression of genes involved in the glyoxylate cycle were repressed in gall tissues (S2 Table). Taking another point of view, because the gene repressed in gall tissues represents the same gene contained at a higher transcription level in the host leaf, it could be speculated that the genes that encode key enzymes in the glyoxylate cycle were up-regulated in the host leaf. The glyoxylate cycle is the gluconeogenesis process that uses fatty acids from lipids as carbon sources during seed germination [21]. The up-regulation of glyoxylate cycle enzymes, such as isocitrate lyase and malate synthase, in the host leaf suggests that the leaf acquired additional glucose from lipids to fulfill the demand for sugar for gall and larval development.

### Phytohormones and stress

The enrichment of BR synthesis-degradation functions in cup-shaped galls suggested that the physiological response to the insect invasion might be regulated by steroid hormones. BRs are synthesized ubiquitously in plant tissues and play essential roles in plant development and stress responses via the activation of transcription and interaction with other phytohormones [22]. BRs targeted the modification of cell wall functions to initiate cell elongation and expansion [23], and thus facilitated the intensive morphological change in plant tissue. In a study of cultured crown galls of *Catharanthus roseus*, two BRs were identified by gas chromatography-mass spectrometry [24]. The galls in *Distylium racemosum* leaves also contained 50–300 fold of BR accumulation [25]. A recent study on *Eucalyptus grandis*, however, showed a down-regulation of BR-related genes in galled leaves [9]. This inconsistency suggests that different signaling pathways are induced in different species.

Increases in endogenous BR synthesis in gall tissue also represented the induction of a stress tolerance mechanism. BRs modulate plant tolerance to abiotic and biotic stresses, such as heat, cold, drought, and salinity [26, 27]. Induction of gall tissues was found to reduce water potential and stomatal conductance in either the leaf or gall tissue. This physiological disorder might alter the content of mineral elements [5, 28] and could lead to drought and salinity stress. Because the genes that respond to abiotic stress were up-regulated in cup-shaped galls (Fig 5), the results of the present study suggested that BRs might also promote tolerance to drought and salinity stress.

The up-regulated expression of RBOH genes suggested that the insect provoked a cellular defense response (S2 Table, [14]. However, the expression of genes encoding pathogenesis-related proteins in the biotic stress response function were suppressed in the gall tissue of *L*. *acuminata* leaves (Fig 5). This might have been caused by the insect and their symbionts. It has been shown that lepidopterans and aphids can secrete glycose oxidase to repress jasmonic acid-associated defensive responses in plant tissues via the salicylic acid signaling pathway [29, 30]. Salicylic acid was also identified in the mucus of slugs and was suggested to inhibit the plant immune response [31]. Therefore, the reprogramed defense signaling pathway of the biotic response in cup-shaped galls should be further examined through the analysis of salicylic and jasmonic acid levels during gall formation and maturation.

Although auxin and cytokinin were suggested to trigger leaf defense mechanisms and induce gall development in plants [9, 10], no enrichment of genes involved in either the IAA or cytokinin synthesis pathway in cup-shaped galls was exhibited (Fig 5). Based on this finding and the observation of increased transcription of auxin-responsive proteins (Fig 7, S3 Table), we suggest that the development of cup-shaped galls might be regulated by auxin derived externally, for example, from the insect larvae. The repression of cytokinin synthase-coding genes and the induction of genes encoding cytokinin signal transduction proteins in gall tissue also indicated exogenous cytokinin production. Previous studies on the transcriptome of insect galls in Myrtaceae showed a higher transcription level of auxin responsive genes in gall tissue with no obvious up-regulation of genes associated with auxin synthesis [10]. Transcriptomic and biochemical study on the apple tree also showed that without the expression of cytokinin-related genes, the accumulation of cytokinin could be strongly induced in insect mined leaves [11]. Taken together, data from these and the present study are consistent with the suggestion that exogenous auxin and cytokinin secreted by insects can manipulate the development of plant tissues [32–35].

## Conclusions

The data from the present research and other available data on the transcriptome of insect galls revealed the genetic regulation and responses of gall tissues. In agreement with previous studies, we demonstrated the modification of functionality of leaves to form gall tissues. Transcriptome research has indicated that the manipulation of gall genes might be induced by auxin and cytokinin secreted by the galler from Hemiptera, Lepidoptera, or Diptera. The present study also present a potential role of steroid hormones (BRs) in gall development. However, more research is needed to fully elucidate this issue and determine if there are unified (or varied) mechanism(s) involved, given the diversity of gallers and host plants.

## Supporting information

S1 Fig Relation of fold change between RNA-seq and qPCR. Data were plotted in the Log2 scale.
(TIF)

S1 Table Homology search of *Litsea acuminata* against 14 plant species in the Plant Database. (XLSX)

S2 Table List and fold change of genes with at least 2-fold change in MapMan functional categories. (XLSX)

S3 Table List and fold change of genes with at least 2-fold change in MapMan phytohormone-associated functional categories. (XLSX)

S4 Table Gene list and the corresponding expressional fold change analyzed by qPCR. (XLSX)

S5 Table Primer sets for qPCR. (XLSX)
