# Supplementary figures and images for: Transcriptome profile of cup-shaped galls in Litsea acuminata leaves
Source: PLoS One. 2018 Oct 24;13(10):e0205265. doi: 10.1371/journal.pone.0205265 (PMC6200225; doi:10.1371/journal.pone.0205265)

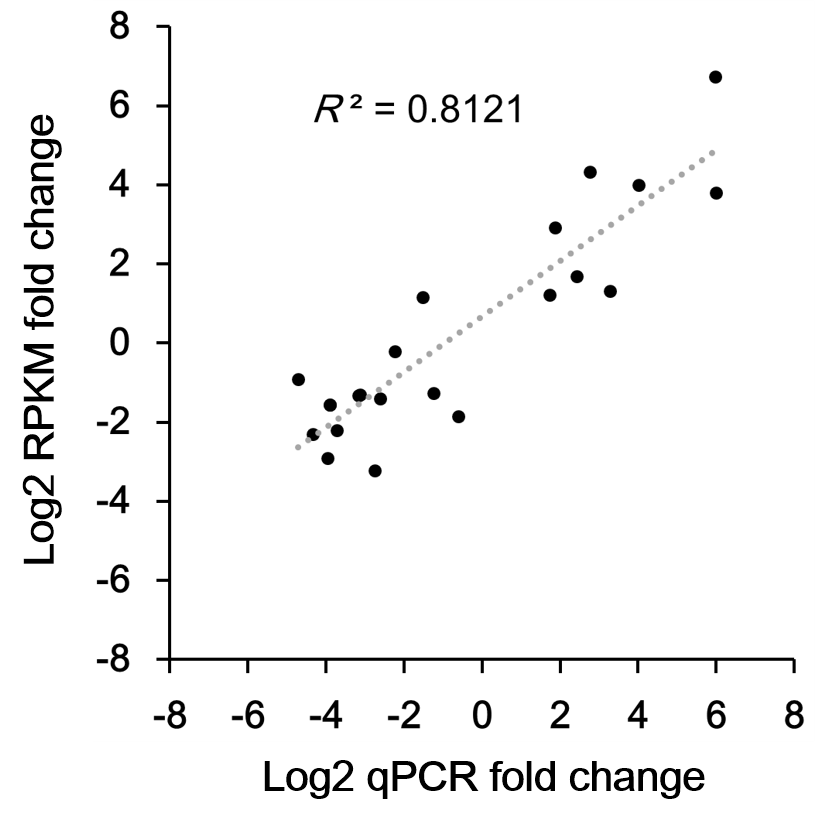

Supplement: S1 Fig — Data were plotted in the Log2 scale. (TIF) [file pone.0205265.s001.tif]
